# Application of Fused Organoid Models to Study Human Brain Development and Neural Disorders

**DOI:** 10.3389/fncel.2020.00133

**Published:** 2020-05-15

**Authors:** Augustin Chen, Zhenming Guo, Lipao Fang, Shan Bian

**Affiliations:** ^1^Institute for Regenerative Medicine, Shanghai East Hospital, School of Life Sciences and Technology, Tongji University, Shanghai, China; ^2^Frontier Science Center for Stem Cell Research, Tongji University, Shanghai, China; ^3^Molecular Physiology, Center for Integrative Physiology and Molecular Medicine (CIPMM), University of Saarland, Homburg, Germany; ^4^Bio-X Institute, Shanghai Jiao Tong University, Shanghai, China

**Keywords:** brain organoid, fusion models, stem cells, brain developement, neural disorder

## Abstract

Human brain organoids cultured from human pluripotent stem cells provide a promising platform to recapitulate histological features of the human brain and model neural disorders. However, unlike animal models, brain organoids lack a reproducible topographic organization, which limits their application in modeling intricate biology, such as the interaction between different brain regions. To overcome these drawbacks, brain organoids have been pre-patterned into specific brain regions and fused to form an assembloid that represents reproducible models recapitulating more complex biological processes of human brain development and neurological diseases. This approach has been applied to model interneuron migration, neuronal projections, tumor invasion, oligodendrogenesis, forebrain axis establishment, and brain vascularization. In this review article, we will summarize the usage of this technology to understand the fundamental biology underpinning human brain development and disorders.

## Introduction

The human central nervous system (CNS) develops from several distinct vesicles into multiple intertwined regions. During this process, a range of migratory streams arise where progenitors generated in one place migrate and integrate into other areas (Marín et al., 2001; Kwan et al., 2012; Clowry et al., 2018; Molnár et al., 2019), and complex networks emerge, neurons branching and projecting across multiple regions (López-Bendito and Molnár, 2003). Besides, the colonization of the embryonic brain by mesodermal derivatives, namely microglia progenitors from the yolk-sac (Rezaie et al., 2005; Monier et al., 2007), and capillaries from the meningeal inner pial lamella (Marín-Padilla, 2012) adds complexity to the development of the human brain. Yet convoluted, the mechanism underpinning the formation of the human CNS is a highly ordered process that needs to be understood.

Human brain organoids are self-organizing three-dimensional stem cell cultures that recapitulate many aspects of the early developing human brain. These organoids include the formation of cortical progenitors with *in vivo*-like morphology and spatiotemporal organization (Eiraku et al., 2008; Kadoshima et al., 2013; Lancaster et al., 2013, 2017; Pasca et al., 2015; Renner et al., 2017) as well as faithful gene expression and epigenome compared to human fetal brains (Camp et al., 2015; Luo et al., 2016; Quadrato et al., 2017; Amiri et al., 2018; Velasco et al., 2019; Trevino et al., 2020). To recapitulate the first developmental stages of the human CNS, human brain organoids overcome many limitations imposed by animal models, providing a unique tool to study early stages of the human brain development under both physiological and pathological conditions (Lancaster and Knoblich, 2014b; Fatehullah et al., 2016). Although there are still many limitations, brain organoids have been applied to model human brain development and disorders since the milestone publication by Eiraku et al. (2008) introducing the model for the very first time.

By comparing the cerebral organoids derived from humans and non-human primates, this technology led to the discovery of human-specific developmental features (Mora-Bermúdez et al., 2016; Otani et al., 2016; Kanton et al., 2019; Pollen et al., 2019). Results from clonal analysis and live-imaging experiments converged on the finding that human cortical progenitors spend more time in a proliferative state (Mora-Bermúdez et al., 2016; Otani et al., 2016). This has been supported by the description of a higher activity of the PI3K-AKT-mTOR pathway—involved in the maintenance of pluripotency—specific to human radial glia from outer subventricular zones (Pollen et al., 2019). These studies provide possible developmental mechanisms to the higher number of neurons observed in the human cortex. When grown from patient-derived induced pluripotent stem cell (iPSC), brain organoids can model some aspects of developmental diseases (Amin and Pasca, 2018), such as microcephaly (Lancaster et al., 2013; Omer Javed et al., 2018; Zhang et al., 2019), macrocephaly (Li et al., 2017), lissencephaly (Bershteyn et al., 2017; Iefremova et al., 2017), autism (Mariani et al., 2015), schizophrenia (Ye et al., 2017), Down syndrome (Xu et al., 2019), and neuronal heterotopia (Klaus et al., 2019). When exposed to viral loads, brain organoids provide new insights into prenatal infections like ZIKV (Cugola et al., 2016; Garcez et al., 2016; Qian et al., 2016). Moreover, brain organoids could be used as *in vitro* screening platform for potential therapeutics and gene editing technologies for the introduction or the suppression of oncogene mutations (Bian et al., 2018). In a nutshell, by reproducing many aspects of the intricate development of the CNS, human brain organoids are used to study the impact of genomic and transcriptomic modifications as well as the exposition to various stress factors on the early development of the human CNS (Schwartz et al., 2015; Lee et al., 2017; Zhu Y. et al., 2017; Belair et al., 2018; Wang et al., 2018).

Throughout the embryonic brain, neural progenitors progressively acquire their spatial identities, a process regulated by the successive actions of patterning centers and transcriptional frameworks (Molnár et al., 2019). When grown without additional patterning molecules, one single organoid can differentiate into various brain regions, including dorsal and ventral forebrain, choroid plexus, hippocampus, and retina (Lancaster et al., 2013). Although some organizing centers were observed in brain organoids (Renner et al., 2017), which are critical for regional brain patterning, most of the spatial identities in organoids appeared in an uncontrolled manner, thereby limiting the study of complex interregional interactions. Application of patterning factors and/or chemicals allows us to pattern brain organoids into different brain regions (Eiraku et al., 2008, 2011; Muguruma et al., 2010, 2015; Nakano et al., 2012; Kadoshima et al., 2013; Sakaguchi et al., 2015; Jo et al., 2016; Qian et al., 2016; Xiang et al., 2019), which provides researchers “LEGO blocks” to establish fused organoid approaches. The development of fused organoids, also called assembloids (Marton and Pasca, 2019), opens a new avenue to investigate interregional dynamics in the embryonic brain. Fusing two brain organoids pre-patterned into different regional identities enables the study of interregional interactions. This organoid fusion technology has already been applied for the study of interneuron migration (Bagley et al., 2017; Birey et al., 2017; Xiang et al., 2017), brain circuits (Giandomenico et al., 2019; Xiang et al., 2019), oligodendrogenesis (Xiang et al., 2017; Kim et al., 2019), and to establish the dorsoventral and anteroposterior axes within forebrain organoids (Cederquist et al., 2019). Organoids made by the fusion approach can help to deconstruct organogenesis by reconstructing the brain piece by piece.

The fusion paradigm should not be limited to the interaction of different brain regions. Any 3D co-culture system involving the assembly of brain organoids with a different tissue can be encompassed in the fusion approaches. This extended definition includes the co-culture of organoids with other cell types, such as endothelial cells (Song et al., 2019; Wörsdörfer et al., 2019) and tumor cells (Ogawa et al., 2018; Linkous et al., 2019; Bhaduri et al., 2020; Zhu et al., 2020). In this review article, we summarize the potential and great diversity of brain organoid fusion and co-culture models in studying human brain development and neural disorders.

## Interneuron Migration

Inhibitory interneurons, mostly GABAergic cells, are mainly derived from two areas of the human embryonic subpallium (ventral forebrain): the medial ganglionic eminence (MGE) and the caudal ganglionic eminence (CGE; Yuste, 2005; Wonders and Anderson, 2006; Bartolini et al., 2013). After production, post-mitotic interneurons migrate tangentially towards the dorsal forebrain, where, they wire and form functional circuits with locally born excitatory neurons. Defects in interneuron proliferation or impeded migration would disrupt the balance between these two types of neurons and are thought to contribute to many neurological and psychiatric disorders, such as schizophrenia, Down syndrome and autism (Wonders and Anderson, 2006; Bartolini et al., 2013; Bagley et al., 2017; Xu et al., 2019).

**Figure 1:**
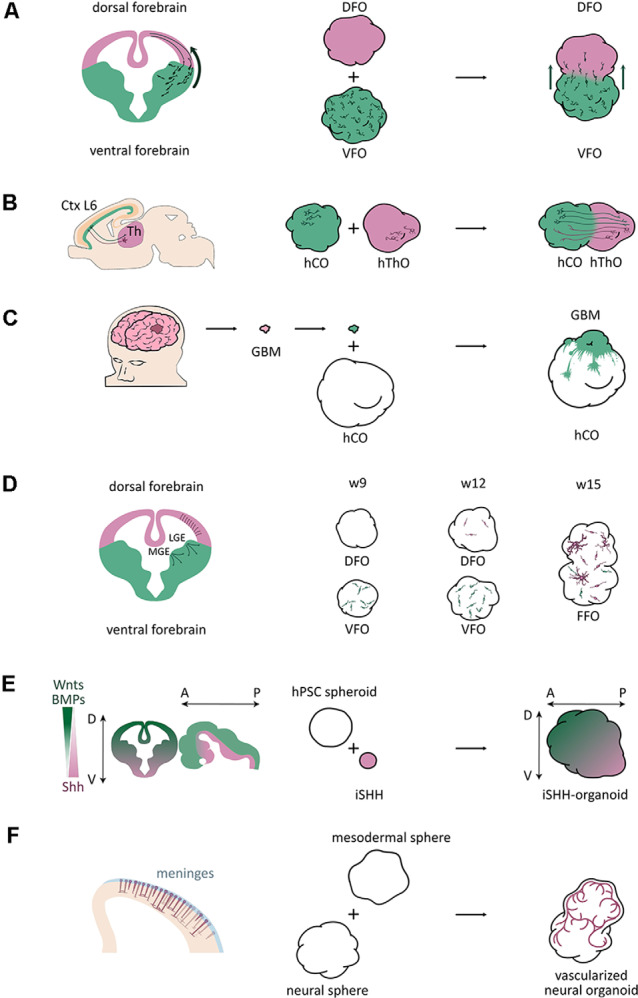
Schematic of application of fused organoid models studying human brain development and disorders. **(A)** A scheme of interneuron migration from ventral to dorsal areas in the mouse brain and fused organoid models (Bagley et al., 2017). DFO, dorsal forebrain organoid; VFO, ventral forebrain organoid. **(B)** A scheme of neuronal projections between the neuronal layer 6 of the cortical plate and the thalamus in the mouse brain and fused organoid models (Xiang et al., 2019). Ctx, cortex; L6, layer 6; Th, thalamus; hThO, human thalamic organoid; hCO, human cortical organoid. **(C)** A scheme of a brain tumor invasion model by fusing patient-derived tumorspheres with brain organoids (Linkous et al., 2019). GBM, glioblastoma multiforme; hCO, human cortical organoid. **(D)** A scheme of the three oligodendrogenic waves in the mouse brain and a scheme of oligodendrogenesis in brain organoids (Kim et al., 2019). MGE, medial ganglionic eminence; LGE, lateral ganglionic eminence; DFO, dorsal forebrain organoid; VFO, ventral forebrain organoid; FFO, fused forebrain organoids; w9, w12, w15, week 9, 12, 15. **(E)** A scheme of axis establishment in the mouse central nervous system (CNS) and a scheme of a SHH gradient-induced forebrain axis establishment of brain organoids (Cederquist et al., 2019). iSHH, induced Sonic hedgehog sphere; D, dorsal; V, ventral; A, anterior; P, posterior. **(F)** A scheme of vascularization in the developing mouse cortex and a scheme of brain organoid vascularization (Wörsdörfer et al., 2019).

Studying interneurons, especially their interaction with excitatory neurons in organoids, would expand our view of these diseases, which are mainly studied using animal models. Although different brain regions, including dorsal and ventral forebrain, can be found in cerebral organoids, they appear in random locations without a stereotyped arrangement (Lancaster et al., 2013, 2017; Lancaster and Knoblich, 2014a), which make it challenging to model interneuron migration in organoids. However, the organoid fusion technique overcame this issue thereby allowing the modeling of human interneuron migration. In 2017, three independent groups generated interneuron migration models using fused organoid approaches (Figure 1A; Bagley et al., 2017; Birey et al., 2017; Xiang et al., 2017). Interneurons were produced in the ventral organoids, and migrated into the dorsal regions after fusion.

The first study using brain organoids to model interneuron migration has been is reported by Birey et al. (2017). Based on a cortical spheroid (hCS) protocol (Pasca et al., 2015), Birey et al. (2017) patterned their organoids into ventral forebrain or subpallium identities by adding WNT inhibitor and Sonic Hedgehog (SHH) agonist during early stages of the differentiation protocol. The results of immunostaining and single-cell RNA sequencing (scRNA-seq) revealed that human subpallium spheroids (hSSs) were composed of various ventral progenitors and GABAergic interneurons, which were also found to be functionally active by patch clamping. After fusing hCSs with hSSs, Dlxi1/2b::eGFP labeled hSS cells were found crossing the fusion boundary, moving further into hCSs, generating elaborated branched morphologies, and forming functional synapses with hCS glutamic neurons. FACS followed by scRNA-seq analysis revealed interneuron transcriptional signatures in eGFP^+^ cells isolated from the hCSs, following previous findings (Zechel et al., 2014). This migration in hSS-hCS fused organoids is consistent with human cortical development. Also, they combined patient-derived iPSCs and the organoid fusion approach to study Timothy syndrome, an L-type calcium channel (LTCCs)-related disorder affecting many organs of the human body including the nervous system. A previous study in mice indicated that LTCCs are associated with interneuron migration (Bortone and Polleux, 2009). Impaired interneuron migration was observed in patient-iPSC derived hSS-hCS, and was rescued by administration of an LTCC blocker.

Bagley et al. (2017) also established a fusion method, but modified from Lancaster’s protocol (Lancaster and Knoblich, 2014a). The WNT inhibitor IWP2 and SHH agonist SAG were applied for organoid ventralization, while CycA, an SHH receptor antagonist, was used to dorsalize organoids (Vazin et al., 2014). In GFP^+^ ventral and dTomato^+^ dorsal fused organoids, a uni-directional cell migration from ventral into dorsal regions was observed. Rarely, migration was observed within the dorso-dorsal fused organoids. The largest proportion of migrated cells was SOX6^+^ MGE cells. Also, COUP-TFII^+^/NR2F2^+^ and SP8^+^ lateral ganglionic eminence (LGE)/CGE cells were observed to migrate into dorsal regions. Slice culture of dorso-ventral fusion organoids and time-lapse imaging analysis revealed characteristic interneuron migratory dynamics. Administration of the CXCR4 antagonist AMD3100 onto organoid slice cultures perturbed the migration, supporting a potential application of the fusion platform for drug screening.

Further, Xiang et al. (2017) generated human MGE organoids (hMGEOs) using an *NKX2-1*::GFP reporter human embryonic stem cell (hESC) line. RNA-seq, scRNA-seq, and chromatin accessibility analysis revealed that hMGEOs exhibited a strong correlation with the transcriptional signature of the MGE tissues from of human fetal brain. By fusing human cortical organoids (hCOs) and hMGEOs, they established hMGEOs-hCOs fusion organoids, named hfMCOs. GFP^+^ but RFP^−^ interneuron progenitors migrated into the neuronal layer of hCOs. Their migration can be inhibited by the application of blebbistatin, a myosin II inhibitor. Besides, functional synapses in hfMCOs were also observed by calcium imaging analysis.

Furthermore, using a dorsoventral organoid fusion model, Yuan et al. (2020) showed that LHX6 is essential for GABAergic interneuron migration. Another study implanted patient iPSC-derived ventralized organoids into the mouse brain to investigate the role of OLIG2 in Down syndrome etiology (Xu et al., 2019). All these studies highlight the great potential of using the organoid fusion approaches to investigate the role of interneuron migration during human brain development and neural disorders.

## Neuronal Projections

Long distance-projections play essential roles in brain functions. According to recent reports, corticothalamic interactions are relevant for sensorimotor interplay, selective attention, and arousal behaviors (López-Bendito and Molnár, 2003). Recently, Xiang et al. (2019) developed a method to form human thalamic organoids (hThOs) and created a model for corticothalamic projection by merging cortical and thalamic organoids (Figure 1B). The expression of the caudal forebrain marker OTX2, ventral thalamic marker DBX1, thalamic marginal zone marker of GBX2, and thalamic marker TCF7L2 significantly increased in the hThOs. Furthermore, scRNA-seq analysis identified specific cell types also found in the human fetal thalamus. After fusing hThOs and hCOs, they found that the axons from both cortex and thalamus reach the other side within 6 days. They also performed whole-cell patch-clamp recordings to examine the functional properties of thalamic neurons. The results revealed that cortico-thalamic neuronal projections might affect the maturation of thalamic neurons. The finding of this study provides a prospect to explore thalamic development and disorders associated with thalamic anomalies, for example, schizophrenia, epilepsy, and autism spectrum disorder.

Another study described the successful formation of neuronal projections between cerebral organoids and the mouse spinal cord (Giandomenico et al., 2019). Giandomenico et al. (2019) applied the air-liquid interface cerebral organoid (ALI-CO) method, which can improve oxygen supply compared to the standard approach, to promote the survival and maturation of brain cells, such as more complex dendrites and dendritic spines. ScRNA-seq indicated that ALI-COs exhibit various neuronal identities correlated with diverse axon morphologies. Interestingly, three-dimensional multi-electrode arrays revealed the highest correlated activity occurred over long distances, suggesting neurons were not limited to nearest-neighbor connections. Using this slice culture approach, they assessed the functionality of subcortically projecting tracts by co-culturing ALI-COs with the spinal cord sections from embryonic mice. Bundles of axon tracts connected to the spinal cord after around 2–3 weeks. Synapses could also be detected between ALI-CO projecting axons and spinal cord neurons. Strikingly, axon tracts could guide mouse muscle contraction when innervated, showing that organoid-derived projections can stimulate spinal cord/muscle explants. This study provides a way to explore neuronal connectivity with both input and output and can be used to study some aspects of neuronal circuit imbalances, degenerative conditions, or spinal cord injury.

Also, Mansour et al. (2018) transplanted human brain organoids into the adult mouse brain. In the chimeric brains, the grafted organoids displayed continuous differentiation and maturation. Moreover, they discovered an extension of axons into various regions of the host brain. Most axon tracts projected out of human organoids into the cortical layers and the corpus callosum. Axons with lower fiber density were observed in the deeper tissues of the host brain, such as the hippocampus and the thalamus, and even the contralateral areas. Furthermore, *in vivo* extracellular recording revealed synaptic connections and neuronal activity within the graft. This study supports the development of cell replacement therapy using brain organoids.

## Brain Tumor

Brain tumors are among the most lethal cancers and are the leading cause of cancer deaths in children under the age of 14 (Ostrom et al., 2016a,b). Their study is limited because of the incompleteness of available laboratory models. Brain organoids provide a novel platform to study brain tumor biology. Brain tumor organoid models were established by genetically introducing clinically relevant mutations into cerebral organoids to mimic brain tumor initiation (Bian et al., 2018; Ogawa et al., 2018). However, some studies implanted or fused patient-derived tumor cells into brain organoids to study brain tumor invasion (Figure 1C; Ogawa et al., 2018; Linkous et al., 2019; Bhaduri et al., 2020) or therapy (Zhu et al., 2020).

Because of its aggressiveness and invasiveness, glioblastoma multiforme (GBM) is the deadliest and most widespread primary brain tumor in adults. Their property to spread through infiltration in the host brain tissue is associated with high resistance and recurrence rate. To understand the mechanism underpinning this invasive phenotype, organoid-GBM fusion models are particularly appropriate. Ogawa et al. (2018) fused tumorspheres from one patient-derived GBM cell line SK2176 with cerebral organoids and found that SK2176 cells invaded and proliferated within the organoid parenchyma rapidly (Ogawa et al., 2018). In another study, Linkous et al. (2019) used brain organoids to provide a “normal” human brain microenvironment for tumors. They introduced cerebral organoid glioma (GLICO) as a model for tumor infiltration by co-culturing glioblastoma stem cells (GSCs) with cerebral organoids. The GLICO model showed that tumor cells deeply invaded the organoid parenchyma, and proliferated within the host tissues. They also observed the formation of an interconnected network of tumor microtubes, which is critical for tumor invasion. Moreover, in the most recent research, Bhaduri et al. (2020) identified an invasive tumor population similar to outer radial glial (oRG) cells using scRNA-seq analysis of patient specimens. They found that PTPRZ1^+^ cells expanded and invaded into the cerebral organoids after engraftment, and showed that PTPRZ1 plays a crucial role in tumor invasion.

Tumor-organoid fusion models have been used for preclinical therapeutic studies. Previously, the Zika virus (ZIKV) has been proven to be a potential oncolytic virus for brain tumor therapy because of its tropism towards tumor cells (Zhu Z. et al., 2017). In further investigation, Zhu et al. (2020) used GBM-brain cerebral organoid (GBM-BCO) models to identify the SOX2-integrin α_v_β_5_ axis as a potential mechanism for ZIKV tropism. Thus, organoid-GSC fusion models could be used as *in vitro* platforms to characterize GBM invasiveness and screen for potential therapeutics.

## Oligodendrogenesis

Oligodendrocytes (OLs) are the myelinating glial cells of the brain and the last type of neural cells to be generated after neurons and astroglial cells during mammalian brain development (Goldman and Kuypers, 2015). Based on the research using animal models, especially rodents, there are three major waves of oligodendrogenesis during forebrain development that have been described. Around embryonic day 12.5 (E12.5) and E15.5 in mice, oligodendrogenesis occurs in the ventral forebrain (Tekki-Kessaris et al., 2001; Kessaris et al., 2006; Chapman et al., 2013). After birth, oligodendrocyte precursors (OPCs) migrate tangentially to the cortex and spread throughout the forebrain. The third wave arises around birth date from the EMX1-expressing precursors in the dorsal forebrain (Kessaris et al., 2006). The dorsally born OPCs migrate locally and replace the OPCs generated from the first two waves. Although oligodendrogenesis has been well studied in animal brains, it remains unclear whether human oligodendrogenesis has any unique features because of the lack of human brain experimental models. The establishment of brain organoids provides a new platform to study human-specific oligodendrogenesis.

In one of the interneuron migration studies, Xiang et al. (2017) observed oligodendrogenesis in hMGEO cultures. They performed chromatin accessibility analysis of hMGEOs by ATAC-seq and found hMGEOs and human MGE shared similar open chromatin regions. Specifically, in ventral organoids, regions of oligodendrocyte-expressing genes were exclusively open. ScRNA-seq analysis showed OLIG1^+^ cells could be found in hMGEOs at day 80, suggesting an earlier oligodendrocyte generation in human MGE than in the cortex.

Another study used dorsoventral fused organoids to study human developmental processes, particularly the dorsally derived oligodendrogenesis (Kim et al., 2019). They generated an OLIG2-GFP knockin hPSC reporter line to visualize OPC generation, and cultured organoids as dorsal forebrain organoids (DFOs), ventral forebrain organoids (VFOs), or fused forebrain organoids (FFOs) to study oligodendrogenesis. Similar to the first two oligodendrogenic waves in the mouse brain, OPCs first appeared in VFO culture. From week 5 to 7, intensive GFP^+^ cells were observed in VFOs. In week 9, a big portion of OLIG2-GFP^+^ OPCs were observed within VFOs (Figure 1D). Only until week 12, the GFP^+^ OPCs were remarkably generated in DFOs, mimicking the third wave of oligodendrogenesis in the mouse brain (Figure 1D). More interestingly, when fusing VFO and DFO into FFO, they found that dorsally born OLs outnumber ventral-derived OLs and become dominant in FFOs after long-term culture, which is also comparable to the oligodendrogenesis in mouse cortex (Figure 1D). Although this study did not find human-specific features, it introduced a novel model to study oligodendrogenesis and myelination-related diseases in a human genetic background.

## Forebrain Axis Establishment

During brain development, topographic structures are generated by gradients of various signaling activities, such as WNT, SHH, and BMP (Petros et al., 2011), that regulate cell fate determination and regional identities. Brain organoids can recapitulate histological features of the human brain, but lack reproducible topographic organization. To overcome this drawback, a recent study established a self-organized dorsoventral and anteroposterior axes in forebrain organoids through an SHH protein gradient (Cederquist et al., 2019). By fusing the forebrain organoid with an inducible SHH-expressing hPSC-derived spheroid, Cederquist et al. (2019) introduced a SHH protein gradient into forebrain organoids (Figure 1E). SHH-patterned brain organoids exhibited *in vivo*-like topography of major forebrain subdivisions. This study opens the possibility to investigate more subtle neurodevelopmental mechanisms and region-specific neural disorders in a single organoid system.

## Brain Organoid Vascularization

During brain development, the vasculature is one of the important niche components for neural stem cells and plays critical roles in neurogenesis (Bjornsson et al., 2015). However, due to a lack of cells from the mesodermal lineage during the brain organoid culture procedure, the absence of vascularization represents one of the major limitations of brain organoid models. There were several attempts to vascularize brain organoids using different approaches. Wörsdörfer et al. (2019) fused mesodermal precursor cell (MPC) aggregates with brain organoids for vascularization (Figure 1F). They found blood vessel-like structures in the brain organoids. Interestingly, these blood vessel-like tissues exhibited typical blood vessel ultrastructures, such as basement membranes, endothelial cell-cell junctions, and microvesicles. Also, they found IBA1^+^ microglia-like cells, which are delivered from MPCs, infiltrating the brain organoids. Another study established hybrid neurovascular spheroids by fusing cortical neural precursor cell (iNSC) spheroids, endothelial cell (iEC) spheroids, and mesenchymal stem cells (MSCs; Song et al., 2019). Furthermore, Cakir et al. (2019) mixed wildtype hESCs with engineered hESCs that ectopically express ETS variant 2 (ETV2) to grow brain organoid. During the culture, vascular-like networks were generated within cortical organoids as ETV2 induces differentiation of hESCs into endothelial cells. All these studies open new doors to improve vascularization of brain organoids. However, it is still far from mimic the *in vivo* vascularization during brain development, and significant improvements still need to be done before obtaining functional vascular networks in organoid cultures.

## Concluding Remarks

Brain organoid fusion technology allows us to study more complicated biology during human brain development and neural disorders using hESCs or patient-derived iPSCs, including interneuron migration, and neuronal projections. Using the same technique, researchers can also study other pathological processes and potential mechanisms, such as corpus callosum deformity. Assembling multiple different brain regions would be the next step towards a more complete human “mini-brain,” which could then be used to study biological mechanisms requiring the interaction of several brain regions *in vitro*. The fusion strategy should not be limited to the fusion of brain tissues as it can be applied to assemble brain organoids with other types of organoids to study the interaction between different organs. For instance, hepato-biliary-pancreatic organogenesis has been modeled using a multi-organoid fusion approach (Koike et al., 2019). In future studies, investigating the interaction between brain organoids and retinal organoids, inner ear organoids, or even intestinal organoids would be of particular interest.

## Author Contributions

AC, ZG, and LF wrote the manuscript. AC and ZG prepared the figures. SB directed the manuscript preparation and wrote the manuscript.

## Conflict of Interest

The authors declare that the research was conducted in the absence of any commercial or financial relationships that could be construed as a potential conflict of interest.
